# Gender Differences and Amputation Risk in Peripheral Artery Disease—A Single-Center Experience

**DOI:** 10.3390/diagnostics13193145

**Published:** 2023-10-07

**Authors:** Viviana Onofrei, Cristina Andreea Adam, Dragos Traian Marius Marcu, Maria-Magdalena Leon, Carmen Cumpăt, Florin Mitu, Doina-Clementina Cojocaru

**Affiliations:** 1 Department of Medical Specialties I, “Grigore T. Popa” University of Medicine and Pharmacy, University Street No. 16, 700115 Iasi, Romania; 2 “St. Spiridon” Clinical Emergency Hospital, Cardiology Department Independence Boulevard No. 1, 700111 Iasi, Romania; 3 Clinical Rehabilitation Hospital, Cardiovascular Rehabilitation Clinic, Pantelimon Halipa Street No. 14, 700661 Iasi, Romania; 4 Clinical Hospital of Pneumophthisiology Iași, Cardiology Department, Doctor Iosif Cihac Street No. 30, 700115 Iasi, Romania; 5 Department of Medical Specialties III, “Grigore T. Popa” University of Medicine and Pharmacy, University Street No. 16, 700115 Iasi, Romania; 6 Academy of Medical Sciences, 030167 Bucharest, Romania; 7 Academy of Romanian Scientists, 700050 Iasi, Romania

**Keywords:** peripheral artery disease, gender, cardiovascular risk, amputation, risk scores, mortality

## Abstract

*Background and Objectives*: Peripheral artery disease (PAD) affects both genders, but the knowledge of clinical and therapeutic aspects particular to each gender has a prognostic value, modulating the risk of amputation and helping to reduce the risk of death or the occurrence of an acute vascular event secondary to optimal management. *Materials and Methods*: We conducted a retrospective, descriptive study that included 652 patients with PAD who were evaluated at “St. Spiridon” Hospital’s Cardiology Department and divided into two groups according to gender: women (100 cases) and men (552 cases). We evaluated demographics, anthropometric data, as well as clinical and paraclinical parameters in the two groups. *Results*: Men had a lower mean age (*p* < 0.001), higher mean BMI (*p* = 0.049) and were more frequent smokers. (*p* = 0.008). Hypercholesterolemia (*p* = 0.026), obesity (*p* = 0.009), concomitant cerebrovascular (*p* = 0.005) and chronic kidney disease (*p* = 0.046) were more common in women, while coronary artery disease (*p* = 0.033) was more common in men. The number of angiographic stenotic lesions (*p* = 0.037) is a statistically significant parameter in our study, with both genders predominantly associated with stenotic lesions. In addition, directly proportional relationships were found between smoking, uric acid, inflammatory markers, and the number of stenotic lesions and thromboses or the ankle–brachial index (ABI). In the subgroup of men, the number of stenotic and thrombosed lesions positively correlated with the ABI value (*p* < 0.001). The presence of more than three cardiovascular risk factors (*p* = 0.001) and serum triglyceride levels (*p* = 0.019) significantly correlated with the number of angiographically detected lesions. We applied several risk scores (PREVENT III, Finnvasc Score, or GermanVasc risk score) in our study group for prognostic purposes, without showing statistically significant differences between genders. Men, rest pain, gangrene, smoking status, the presence of more than three cardiovascular risk factors, or a serum HDL-cholesterol level below 40 mg/dL (*p* < 0.001 for all parameters) are independent predictors associated with amputation in our study group. *Conclusions*: In our study, we demonstrated that several clinical–paraclinical particularities guide the diagnosis, providing the clinician with prognostic and therapeutic tools to choose the optimal management with maximum benefits.

## 1. Introduction

PAD is one of the leading cardiovascular causes associated with a high risk of morbidity and mortality, affecting more than 200 million adults globally according to recent population studies [1]. Still under-diagnosed and under-treated, patients with PAD have a high cardiovascular risk, which can cause the occurrence of a potentially fatal acute vascular event, which hinders both the patient and their family with multiple long-term medical, economic and psycho-social implications [2]. Epidemiological studies have shown that the prevalence of PAD has doubled in the last three decades, secondary to increased life expectancy, increased prevalence of cardiovascular risk factors, or the development of easily implemented screening programs even in developing countries. A significant percentage of PAD patients is associated with multivessel disease, the association of PAD with coronary atherosclerotic disease tripling the risk of death in this category of patients [3,4,5].

Specialized clinical studies published in recent years have highlighted several diagnostic, therapeutic, and prognostic differences between the two genders. Thus, compared to men, women have a marked functional decline, associated with a decreased quality of life and, therefore, a higher risk of a potentially fatal acute vascular event [6,7,8]. Women with PAD have an 18% lower level of physical activity compared to men, which justifies the extension of clinical research in this special population to halt the associated functional decline and implicitly increase quality of life, highlighting important social aspects in the management of patients with PAD [8,9].

The sex-dependent response to various vascular stressors acting in a similar structural and functional arterial substrate induces diverse clinical manifestations of peripheral vascular disease, with a predominance of aortic aneurysm and dissection in men and predominant lower vascular axis involvement in women (frequently women-specific plurivascular involvement) [6,10].

In terms of therapeutic management, gender-dependent differences in prognostic role have been reported. Women are less frequently treated with lipid-lowering medication; therefore, a lower percentage of women reach the therapeutic targets recommended by clinical practice guidelines. A similar pattern has been observed among patients receiving antiplatelet medication or revascularization (with a clear decrease in the percentage difference between genders) [6].

A significant percentage of PAD patients have severe lesions, which increases the risk of a potentially fatal acute vascular event. Several groups of investigators have developed various risk scores with a high predictive value in assessing the risk of amputation at various time intervals (30 days, 5 years) or depending on the type of management (surgical or interventional). The use of these modern quantification tools has both a therapeutic and prognostic value, contributing to the choice of a therapeutic strategy with a maximum benefit for each patient [11,12].

In this study, we aimed to identify the clinical, anatomical, and biological gender differences among patients with PAD, which could prove relevant for optimizing therapeutic approaches and improving the short-term and long-term prognosis of this common vascular disease. We also aimed to investigate the usefulness of risk scores that predict the risk of amputation according to gender.

## 2. Materials and Methods

### 2.1. Study Design

**Figure 1:**
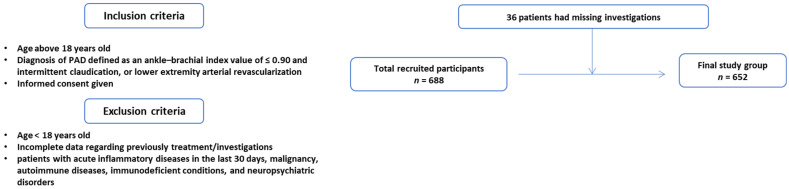
Flow chart of the study group.

We conducted a retrospective, descriptive study on 688 consecutive patients diagnosed with PAD between January 2012 and November 2014, who were examined at the “St. Spiridon” Hospital’s Cardiology Department. Due to incomplete medical data (angiographic examination or biological parameters), 36 individuals were eliminated from the initial study group. As a result, the final research group included 652 individuals with PAD who were comprehensively examined (Figure 1). Inclusion criteria were being above the age of 18, having a clear diagnosis of PAD according to the clinical standards of the European Society of Cardiology [13,14,15] (an ankle–brachial index (ABI) value of ≤0.90 and intermittent claudication, or lower extremity arterial revascularization), and signing an informed consent. Exclusion criteria included being under the age of 18, lack of consent to participate in the study, or having inadequate medical data. We also excluded from the study group patients with acute inflammatory diseases in the last 30 days, malignancy, autoimmune diseases, immunodeficient conditions, and neuropsychiatric disorders.

The existence of symptoms indicative of PAD was evaluated in the absence of a confirmed diagnosis of PAD obtained using vascular Doppler ultrasound or peripheral angiography. Intermittent claudication (IC), paresthesia in the lower limbs, a lack of pilosity, chilly and pale skin, petechiae, or the appearance of dermatitis or ulcers caused by diminished vascularity are all symptoms of PAD.

### 2.2. Measurements

#### 2.2.1. Comorbidities and Laboratory Data

In our study, we included demographic, anthropometric, and paraclinical (biological, imaging) parameters. The existence of significant cardiovascular risk factors such as hypertension [13], diabetes mellitus [14], dyslipidemia [15], smoking, obesity, and sedentary lifestyle was indicated by anamnesis. Smoking was assessed in “pack years”, with a pack-year equaling 20 cigarettes smoked daily for one year [16]. Chronic kidney disease was assessed according to KDIGO guidelines [17].

The observation charts included medical data on demographics, personal medical history, cigarette and alcohol usage patterns, and chronic medication. Body mass index (BMI) was determined as the ratio of weight (kg) to height (m^2^). According to international norms, the body weight of patients involved in the study was assessed using a calibrated medical scale. Measurements were taken on an unweighted basis for each subject, without the use of garments that may cause considerable weight variations. All patients included in the trial had their blood pressure profile evaluated, with the primary parameters utilized in the statistical analysis being systolic blood pressure (SBP, mmHg), diastolic blood pressure (DBP, mmHg), and pulse pressure (PP, mmHg).

Lipid (total cholesterol, low-density lipoprotein cholesterol, high-density lipoprotein cholesterol, triglycerides) and carbohydrate profile (serum glucose), inflammatory markers (serum fibrinogen, hs-CRP), and renal function (serum creatinine, urea) parameters were also assessed.

#### 2.2.2. Transthoracic Echocardiography

Transthoracic echocardiography was conducted during both the initial and subsequent imaging evaluations performed during hospitalization, following European standards (European Association of Cardiovascular Imaging) for the functional and morphological assessment of the heart [18]. The same expert cardiologist performed all imaging exams with the same echocardiograph (Toshiba Aplio 500 Series, Toshiba Medical Systems Corporation, Tawara, Tochigi, Japan). The LV ejection fraction (LVEF) was calculated using the Simpson biplane technique to examine the LV systolic function.

#### 2.2.3. Angiography

The gold standard for diagnosing and treating PAD is peripheral angiography. All patients were provided with information about the risks and potential complications associated with the minimally invasive procedure before the intervention. Biological samples were obtained from all patients obtained (particularly renal function, complete blood count, blood group, and hemostasis parameters), and a venous line was fitted. It was suggested that diabetic individuals taking Metformin stopped taking this medication 24 h before the procedure and resumed taking it after 48 h to reduce the risk of related nephrotoxicity. Angiography was the standard method utilized in all interventional cardiology centers [19,20]. The vascular system was visualized using contrast media injections at the aorto-iliac, femuro-popliteal, and infra-popliteal levels. This procedure was carried out in compliance with clinical procedures in patients with stenotic lesions who needed interventional revascularization. The same cardiologist performed all angiograms.

The Global Limb Anatomic Staging System (GLASS) was used to determine the severity of atherosclerotic lesions and their appropriateness for interventional revascularization [21]. The WIfl classification was used to determine the risk of amputation, taking into account the presence of trophic lesions, ischemia, or related leg infections. The WIfl classification considers the presence of three key components: trophic lesions, symptoms of ischemia, and infection in the foot (rated from 0 to 3 points depending on severity). The trophic lesion is quantifiable as follows: 0—no ulcer, no rest discomfort; 1—tiny, superficial ulcer placed distal or at the level of the foot, without gangrene; 2—deep ulcer with exposure of bone, joint, or tendon, maybe with toe gangrene; and 3—deep, widespread calf ulcer, maybe with calcaneal or extensive gangrene. Classification of ischemia-associated lesions: 1—ABI between 0.6 and 0.79, ankle BP between 70 and 100 mmHg, and halo BP between 40 and 59 mmHg; 2—ABI between 0.4 and 0.59, ankle BP between 50 and 70 mmHg, and halo BP between 30 and 39 mmHg; 3—ABI below 0.4, ankle BP below 50 mmHg, and halo BP below 30 mmHg. Foot infection was measured in the following way: 0 means no indications or symptoms of infection; 1 indicates local cutaneous and subcutaneous cellular tissue infection; 2 indicates deeper local infection than the preceding group; and 3 indicates pre-existing systemic inflammation [22].

### 2.3. Risk Scores

The PREVENT III score for PAD patients undergoing infra-inguinal bypass surgery is used to predict the risk of survival without amputation by considering 5 parameters: dialysis requirement (4 points), tissue loss (3 points), age over 75 years (2 points), hematocrit below 30% (2 points), and concomitant presence of coronary artery disease (1 point). Summing the scores mentioned, patients are divided into 3 distinct risk categories: low (below 3 points), medium (4–7 points), and high (above 8 points) [23].

The Finnvasc score predicts the risk associated with the need for major amputation as well as the risk of death in patients with critical infra-inguinal injuries treated intravenously. The risk score assesses 4 distinct parameters (diabetes, coronary artery disease, gangrene, and urgency of surgery), the risk of death or amputation at 30 days being directly proportional to the score value [12,24,25].

We also assessed the risk of death or amputation at 5 years among patients with chronic critical ischemic lesions based on the GermanVasc risk score. The parameters used to calculate the risk are age (over 80 years—11 points, 71–80 years—6 points, 61–70 years—3 points), vascular dementia—7 points, dementia—6 points, dialysis—6 points, gangrene—4 points, neoplasms—4 points, heart failure—3 points, hydroelectrolyte disturbances—3 points, renal failure—2 points, arrhythmias—2 points. Based on the above-mentioned scores, patients were divided into 3 risk categories: low (0–12 points), medium (13–15 points), and high (16–41 points) [26].

### 2.4. Statistical Analysis

We used the Statistical Package for the Social Science (SPSS) statistics software (version 26 for Windows; SPSS Inc., Chicago, IL, USA) for statistical analysis of the parameters presented above. The results were reported as mean ± standard deviation (SD) for metric variables or frequency, and percentages for categorical parameters. We tested the normal distribution of the data using the Kolmogorov–Smirnov test, and then we applied an independent *t*-test to determine the significance of the differences between the groups (men vs. women) for all normally distributed continuous variables and the Mann–Whitney *U* test for those not respecting the normal distribution law; this also applied to the categorical variables. To assess the association between the studied variables, Pearson (for continuous, normally distributed variables) and Spearman (for categorical variables) correlation coefficients were used. Receiver operating characteristic (ROC) analysis as well as univariate and multivariate analyses were performed to identify clinical predictors in patients with amputations. In terms of the results of the statistical power analysis, we calculated that a general population of 356 cases (80% power for a 95% confidence interval) was required (in order not to have an under-represented group of women, we opted to enroll more patients). A *p*-value of ≤0.05 was considered to be statistically significant.

### 2.5. Ethics

The study was approved by the Ethics Committee of the “Grigore T. Popa” University of Medicine and Pharmacy Iasi and of “St. Spiridon” Clinical Emergency Hospital and was conducted according to the Helsinki Declaration. All patients signed an informed consent statement, which mentioned that the results would be used for research purposes.

## 3. Results

In our study, we analyzed a cohort of 652 patients diagnosed with PAD (84.7% men, with a mean age of 66.46 ± 10.47 years old). Based on gender, we divided the initial study group into two groups: women (first group: 100 cases, 15.3%) and men (second group: 552 cases, 84.7%).

We evaluated various demographic, hemodynamic, biochemical, imaging and angiographic parameters, which are presented in Table 1. In addition to these, we quantified the presence of major cardiovascular risk factors as well as clinical elements associated with the severity of PAD. The mean age of women was higher, with statistical significance (70.27 ± 10 vs. 65.79 ± 10.63 years, *p* < 0.001). Regarding the analyzed anthropometric parameters in our study, BMI (*p* = 0.049) and abdominal circumference (*p* = 0.031) were statistically significant. In terms of vital parameters, the mean value of systolic BP was higher among the first group analyzed, but without statistical significance (*p* = 0.704).

We assessed the presence of major cardiovascular risk factors, and the percentage of patients who were associated with more than three cardiovascular risk factors was 31.0% in the first group (31 patients) and 33.70% (186 cases) in the second group. When comparing the two groups, smoking (*p* = 0.008), hypercholesterolemia (*p* = 0.026), obesity (*p* = 0.009), chronic kidney disease (*p* = 0.046), and the concomitant presence of cerebrovascular disease (*p* = 0.005) or coronary artery disease (*p* = 0.033) are statistically significant comorbidities in our study. The number of cardiovascular risk factors was quantified, with patients in both groups being predominantly associated with two cardiovascular risk factors (40% vs. 35.93%).

Women were associated with more severe atherosclerotic lesions, with the highest number of cases being staged in Rutherford class 5 (32.0%), compared to men where the main percentage category was in Rutherford class 4 lesions (34.12%). Among the biological parameters, statistical analysis revealed significant mean differences in total cholesterol (*p* = 0.004), LDL-cholesterol (*p* = 0.018), HDL-cholesterol (*p* = 0.001), serum triglycerides (*p* = 0.019), or inflammatory markers (serum fibrinogen: *p* = 0.019; hs-CRP: *p* = 0.041). Among the clinical parameters evaluated, the presence of a cardiac murmur (*p* = 0.042) or a carotid murmur (*p* = 0.021) are significant parameters in relation to gender, according to the weight of pluri-vascular involvement detailed above.

**Figure 2:**
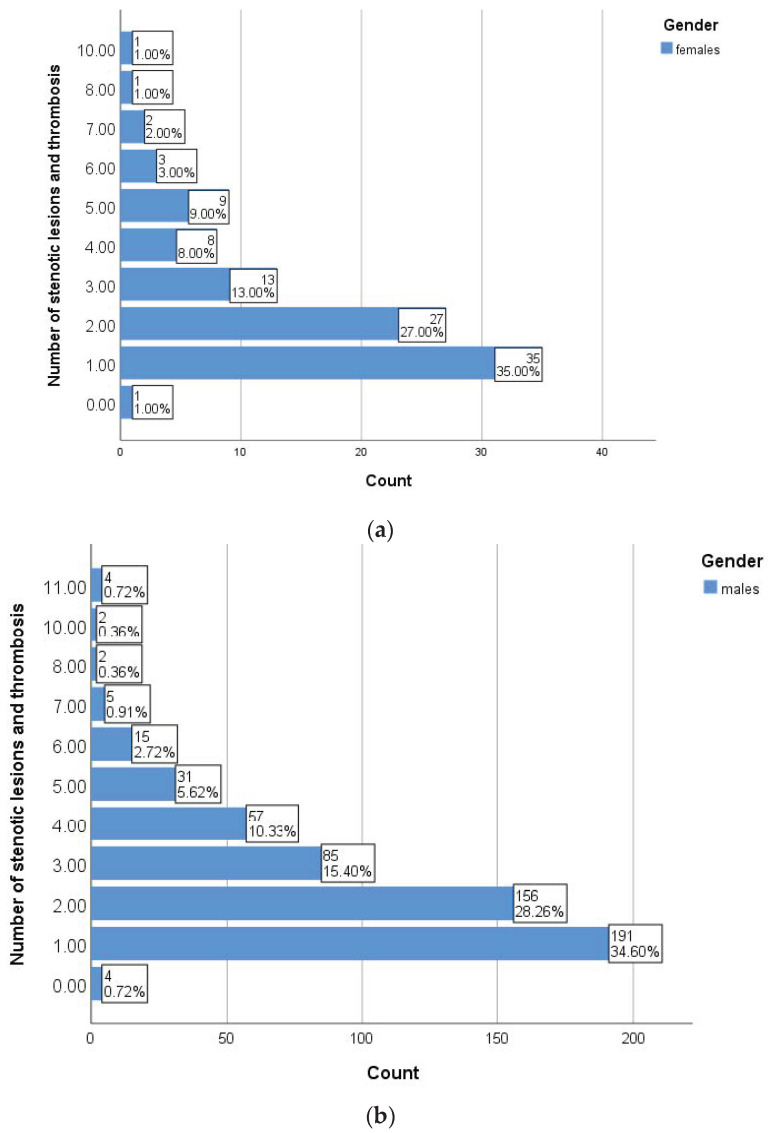
Burden of stenotic lesions in women (**a**) and men (**b**).

Among the angiographic parameters, we paid particular attention to the number of stenotic and thrombotic lesions identified, with more than 30% of patients in both groups associated with a single significant lesion (35% vs. 34.60%, *p* = 0.037) at the time of angiographic evaluation (Figure 2).

**Figure 3:**
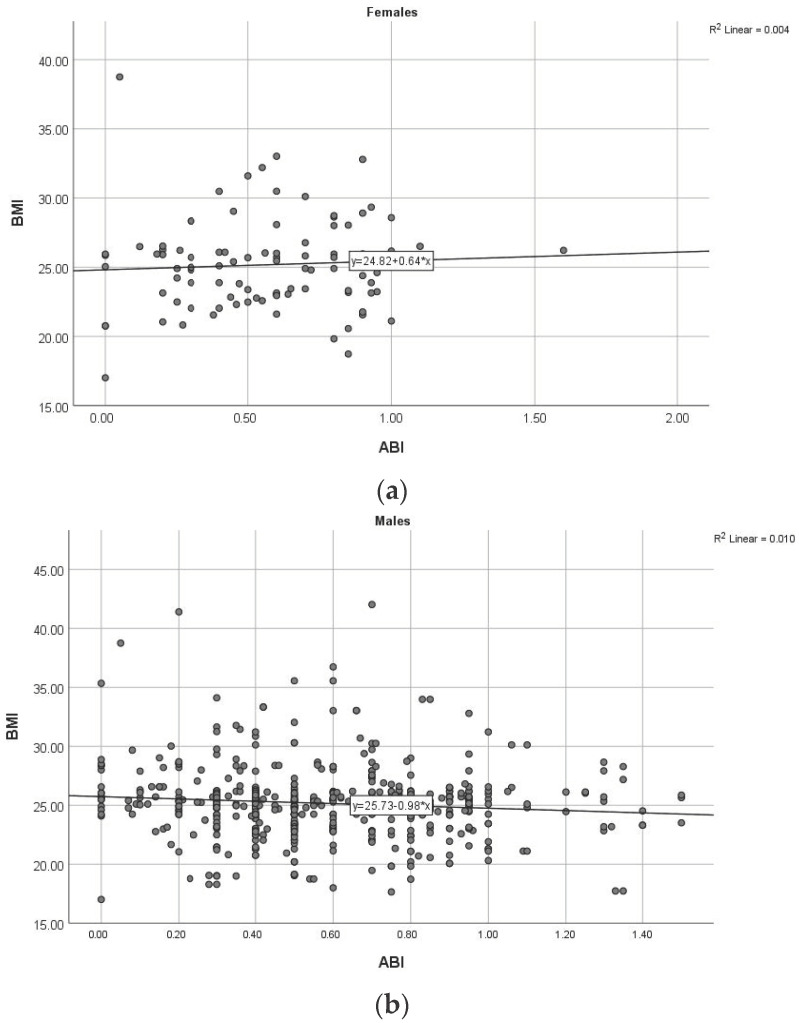
Correlation between ABI and BMI in women (**a**) and men (**b**). (ABI: ankle–brachial index, BMI: body mass index).

We performed a series of correlations between the number of stenotic lesions and thromboses, ABI and various clinical parameters assessed in patients with PAD enrolled in the study. As shown in Table 2, smoking and inflammatory markers showed statistically significant correlations with the aforementioned parameters independent of gender, while uric acid positive correlations were only found in women. In the subgroup of men, statistical analysis revealed a statistically significant correlation between the number of stenotic and thrombosed lesions and the ABI value (r = −0.153, *p* < 0.001) (Figure 3). The presence of more than three cardiovascular risk factors (*p* = 0.001) and serum triglyceride levels (*p* = 0.019) are significantly correlated with the number of angiographically detected lesions. The number of stenotic lesions was a statistically significant parameter between genders (*p* = 0.037), with both genders predominantly showing a lesion in the angiographic evaluation.

**Figure 4:**
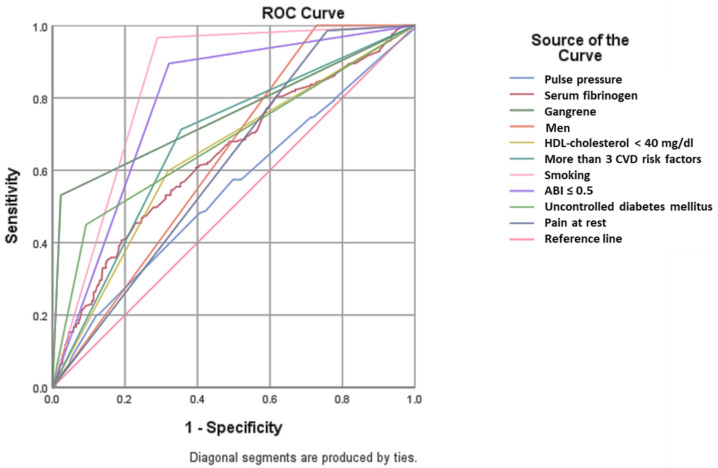
ROC analysis. Predictors for amputation. (HDL: high density lipoprotein; CVD: cardiovascular disease; ABI: ankle–brachial index).

We analyzed the presence of clinical predictors in patients with PAD and amputations (independent of gender) (Figure 4 and Table 3 and Table 4). In our study group, amputations occurred preferentially in men (area under the curve < AUC > 0.636, *p* < 0.001), smokers (AUC = 0.838, *p* < 0.001) with an ABI value of less than 0.5 (AUC = 0.787, *p* < 0.001) and more than three CVD risk factors (AUC = 0.679, *p* < 0.001).

Univariate and multivariate statistical analysis identified clinical (rest pain, gangrene), cardiovascular (men, smoking, dyslipidemia) or paraclinical (serum fibrinogen or low HDL-cholesterol, severe obstruction defined by an ABI less than 0.5) predictors for amputation in patients with PAD, which are presented in the table below (Table 4).

We calculated in our group of patients various risk scores associated with the risk of amputation in patients with PAD treated with an intervention or surgically. Depending on the type of score used and the therapeutic intervention carried out, we formed several subgroups, which are presented below:

- **PREVENT III risk score**: Of the patients undergoing surgical revascularization, 42 women (87.5%) and 297 men (92.81%) were treated in an intervention via infra-inguinal bypass. The mean value of the score was approximately equal in the two statistically analyzed groups (2.89 ± 0.18 vs. 2.63 ± 0.09, *p* = 0.882), discretely higher in the patients of the first group taking into account older age and the presence of a higher percentage of coronary artery disease.
- **Finnvasc Score**: A small percentage of the patients included in the study were treated with an intervention, and of these, 60% of women (3 cases) and 65.11% of men (28 cases) had associated critical injuries requiring emergency treatment. The mean score calculated in this subgroup of patients was higher among men but was not statistically significant (*p* = 0.788).
- **GermanVasc risk score**: Analyzing the subgroup of patients with critical injuries, we found approximately equal scores in both subgroups, without statistical significance (13.18 ± 1.59 vs. 12.89 ± 1.44. *p* = 0.182).

## 4. Discussion

PAD is a cardiovascular disease with multiple gender-dependent features; therefore, paying special attention to gender-specific aspects with diagnostic, therapeutic, and prognostic roles is vital [27]. Based on epidemiological studies published in the literature that highlight sex differences in cardiovascular pathology as well as associated prognostic and therapeutic implications, we decided to comparatively analyze PAD in terms of sex. Thus, we aimed to outline the profile of the women and men with PAD. The knowledge of some particular elements of pathology in the two sexes has scientific and practical value on a daily basis, given the increasing prevalence of cardiovascular risk factors, many predisposing to PAD. In the case of PAD patients, risk stratification by gender is not introduced in the clinical guidelines of the ESC. The clinical studies published thus far in the literature, which highlight the different evolution of PAD patients according to gender and its role in stratifying the risk of an acute vascular events, are recent (within the last 5 years). We consider it a good time to provide more clinical arguments useful to the clinician, leading to the development of future guidelines. By analyzing a broad spectrum of parameters, we outlined two distinct clinical pictures, which help the clinician in their day-to-day work and allow therapeutic strategies to be customized.

Literature data highlight that the clinical picture of women with PAD is dominated by the presence of atypical claudication or even a lack of symptoms at the time of diagnosis. Sexual dimorphism and gender-dependent variations associated with responses to different stressors are aspects involved in the pathophysiological gender differences found in PAD. Sex chromosomes play an important role in vascular structural and functional damage that is predisposed to atherosclerosis [28,29,30]. Based on the concept that the onset of menopause and reduced estrogen production increases cardiovascular risk in women [31], Taddei et al. [32] demonstrated that age-associated endothelial dysfunction is attenuated before menopause in normotensive or hypertensive patients compared to men.

Clinical studies published thus far in the literature have also reported gender-dependent differences in the proportion of cardiovascular risk factors involved in the development and progression of PAD. Thus, smoking was more frequently associated with PAD in men, similar to the results of our study. In our study, the percentage of patients who smoked doubled among men (*p* = 0.008), which justifies the significant percentage of men enrolled in the study, considering that smoking is the main risk factor associated with the development and progression of PAD. The negative impact of smoking on the onset or progression of PAD is independent of gender, with data in the literature associating a high risk with up to 30 years after smoking cessation, a risk dependent on quantity and duration [33,34].

In a similar clinical study, Hiramoto et al. [35] showed in patients with ABI values below 0.9, the presence of the same gender-independent risk factors such as older age, black race, smoking, diabetes, hypertension, dyslipidemia, elevated hs-CRP levels and concomitant coronary artery disease. However, advanced statistical analyses revealed statistically significant correlations between age, hs-CRP, coronary heart disease and diabetes in men. In our study, hs-CRP was statistically significantly correlated with both the number of stenotic lesions and the ABI value independent of gender (*p* < 0.005 for all correlations).

Diabetes mellitus is the main risk factor for progression to severe PAD, with chronic limb-threatening ischemia or even amputations [36,37,38,39]. In our study, independent of gender, we found a higher prevalence of diabetes mellitus among women, associated with a higher proportion of injuries (with a severity of more than 50% and 70%), as well as a higher proportion of cases with severe injuries or amputations. Hypertension is another cardiovascular risk factor negatively affecting the prognosis of PAD patients, with a directly proportional relationship between the severity of blood pressure values and increased risk of developing PAD among women [1,40]. Additionally, Kannel et al. [41] demonstrated that the relationship between hypertension and intermittent claudication is more pronounced among women. Different from the results presented above, in our study, the percentage of patients with hypertension was higher among men (45% vs. 48.91%), with no associated statistical significance.

Similar to hypertension, dyslipidemia had a higher prevalence among women with PAD, while men were associated with lower serum LDL-cholesterol levels, independent of age. Clinical research in the field has also shown higher serum levels of LDL particles associated with a higher risk of developing PAD over time among younger women [42,43,44]. In our cohort of PAD patients, the prevalence of dyslipidemia was higher among women (62.0% vs. 52.17%), and a significant difference was found among patients with serum total cholesterol values above 200 mg/dL (51% vs. 36.13%). Similar to the results reported by other clinical studies, in our study, we identified higher mean serum LDL-cholesterol values among women (*p* = 0.018).

In addition to the traditional risk factors analyzed above, gender-dependent differences were also reported for non-traditional elements involved in the pathogenesis of PAD, such as autoimmune diseases more commonly associated with the clinical picture of women or chronic kidney disease. In the case of the latter, cardiovascular risk increases independent of gender, with different investigators demonstrating a 50% increase in the incidence of PAD in women over 70 years old compared to that in a similar group of men [45,46,47]. In our study, chronic kidney disease was present in a representative percentage in both groups analyzed, being a statistically significant parameter with a predictive role in various risk scores presented in the previous section.

The biological picture of PAD patients differs according to sex, with literature data highlighting the presence of elevated serum triglycerides, hs-CRP, or tobacco metabolites in women, while men were predominantly associated with higher serum fibrinogen and NT-proBNP values [36]. Similar issues were highlighted in our study. Mean serum hs-CRP values were higher among women (*p* = 0.041), while a statistical analysis revealed higher mean serum values for fibrinogen in men enrolled in the study (*p* = 0.049). hs-CRP is an inflammatory marker with multiple therapeutic and prognostic implications in patients with cardiovascular disease, as we also highlighted in our study [48,49].

Gender-dependent differences have also been reported in the associated risk of death or acute vascular events. Pabon et al. [50] highlighted that women with PAD have a higher risk of death or acute cardiovascular events compared to a similar group of women without PAD. Data in the literature on sex differences in the risk of cardiovascular death from any cause are conflicting. Morrell et al. [51] and Sigvant et al. [52] found a higher risk among men with PAD. In terms of the associated risk of developing an acute vascular event, no gender-dependent differences were found, but women were associated with a higher risk of above-knee amputation than men [42]. The presence of critical ischemic lesions is accompanied by a high risk of amputation of up to 40% within 12 months, with reported mortality rates being similar in both sexes [53,54]. In our study, no statistically significant differences in amputation rates were reported, with approximately 30% of patients in both sublots at risk (*p* = 0.854).

A significant percentage of PAD patients have severe lesions, which increase the risk of a potentially fatal acute vascular event. Several groups of investigators have developed various risk scores with a high predictive value for assessing the risk of amputation at various time intervals (30 days, 5 years) or depending on the type of management (surgical or interventional). The use of these modern quantification tools has both a therapeutic and prognostic value, contributing to the choice of a therapeutic strategy with maximum benefit for each patient.

Our study has some follow-up-related limitations. Additional factors that could affect the outcomes include the research group’s heterogeneity or the possible danger of including individuals who have increased blood CRP levels as a result of related infections. Records without access to medical information were disregarded. This was carried out to minimize the risk of misclassification, introducing a limited risk of selection bias.

## 5. Conclusions

In our study, several clinical and therapeutic peculiarities of PAD patients according to gender were demonstrated to facilitate the diagnostic algorithm and the choice of optimal therapeutic strategy to improve long-term prognosis and decrease the risk of associated morbidity and mortality. Dyslipidemia, association with chronic kidney disease, pluri-vascular damage or evidence of more severe lesions are particular to women, while smoking, diabetes mellitus, and high serum fibrinogen levels are more commonly associated with men. Among men with PAD, the presence of more than three cardiovascular risk factors correlates statistically significantly with the number of stenotic lesions and thromboses. Although the assessment of long-term prognosis is facilitated by the use of risk scores with a high predictive value for amputation risk, we have not highlighted any differences between the two genders that suggest an additional benefit in their use. Our study results demonstrate the relevance of several clinical and paraclinical parameters for a gender-specific approach to PAD patients, targeting the maximum vascular benefit of each individual case.

## Figures and Tables

**Table 1:** Demographics, anthropometric parameters, vitals and biological data.

Parameter	Total Group (n = 652)	Women (n = 100)	Men (n = 552)	*p*
**Demographics**				
Age	66.46 ± 10.47	70.27 ± 10	65.79 ± 10.63	<0.001 ^#^
Area of residence (urban)	273 (41.9%)	49 (49%)	224 (40.58%)	0.124 ^†^
**Anthropometric data**				
Height, m	1.92 ± 6.4	1.67 ± 0.05	1.95 ± 6.75	0.325 ^#^
Weight, kg	75.94 ± 9.15	78.69 ± 10.64	91.38 ± 8.92	0.044 ^#^
BMI, kg/m^2^	26.21 ± 3.01	25.09 ± 3.3	27.14 ± 3.05	0.049 ^#^
Abdominal circumference, cm	98.85 ± 10.16	95.14 ± 8.79	103.58 ± 11.05	0.031 ^#^
**Vitals**				
HR, bpm	74.12 ± 13.96	77.07 ± 15.46	76.22 ± 13.88	0.825 ^#^
Systolic BP, mmHg	141.93 ± 14.89	151.30 ± 14.92	141.91 ± 14.62	0.704 ^#^
Diastolic BP, mmHg	80.15 ± 7.66	88.40 ± 7.74	80.13 ± 7.56	0.750 ^#^
Mean BP, mmHg	100.74 ± 8.93	109.70 ± 9.15	100.72 ± 8.75	0.981 ^#^
Pulse pressure, mmHg	73.56 ± 12.99	83.75 ± 15.44	74.19 ± 13.62	0.772 ^#^
**Cardiovascular risk factors and comorbidities**				
Smoking	435 (66.72%)	35 (35.0%)	400 (72.46%)	0.008
Smoking (pack-years)	23.69 ± 18.43	10.69 ± 15.99	25.89 ± 19.10	0.017 ^#^
Dyslipidemia	350 (53.68%)	62 (62.0%)	288 (52.17%)	0.070 ^†^
Diabetes mellitus	213 (32.67%)	29 (29.0%)	184 (33.33%)	0.630 ^†^
Hypercholesterolemia (>200 mg/dL)	267 (40.95%)	51 (51.0%)	216 (36.13%)	0.026 ^†^
Hypercholesterolemia (>250 mg/dL)	67 (10.28%)	13 (13.0%)	54 (9.78%)	0.330 ^†^
HDL-cholesterol < 40 mg/dL	244 (37.42%)	16 (16.0%)	116 (21.01%)	0.101 ^†^
LDL-cholesterol > 130 mg/dL	276 (42.33%)	48 (48.0%)	228 (41.30%)	0.212 ^†^
Hypertriglyceridemia	35 (5.37%)	11 (11.0%)	24 (4.34%)	0.137 ^†^
Overweight	51 (70.8%)	24 (24.0%)	26 (4.71%)	0.009 *
Obesity class I	18 (25.0%)	10 (10.0%)	8 (1.44%)	
Obesity class II	3 (4.2%)	2 (2.0%)	1 (0.18%)	
Hypertension	315 (48.31%)	45 (45.0%)	270 (48.91%)	0.696 ^†^
Number of risk factors				
0	16 (2.5%)	1 (1.0%)	15 (2.72%)	0.478 ^†^
1	180 (27.6%)	28 (28.0%)	152 (27.59%)	
2	238 (36.6%)	40 (40.0%)	198 (35.93%)	
3	156 (24.0%)	25 (25.0%)	131 (23.77%)	
4	45 (6.9%)	6 (6.0%)	39 (7.08%)	
5	16 (2.5%)	-	16 (2.90%)	
Cerebrovascular disease	51 (7.82%)	11 (11.0%)	40 (7.24%)	0.005 ^†^
Coronary artery disease	294 (45.09%)	38 (38.0%)	256 (46.37%)	0.033 ^†^
Chronic kidney disease	70 (10.73%)	21 (21.0%)	49 (8.87%)	0.046 ^†^
**Rutherford classification**				
Class 3	106 (16.3%)	22 (22.0%)	84 (15.25%)	
Class 4	213 (32.7%)	24 (24.0%)	188 (34.12%)	
Class 5	205 (31.4%)	32 (32.0%)	173 (31.40%)	
Class 6	128 (19.6%)	22 (22.0%)	82 (14.86%)	
**Biological data**				
Total cholesterol. mg/dL	198.47 ± 46.57	207.69 ± 46.70	193.14 ± 45.74	0.004 ^#^
LDL-cholesterol. mg/dL	126.82 ± 40.30	142.44 ± 40.80	125.74 ± 40.03	0.018 ^#^
HDL-cholesterol. mg/dL	41.65 ± 10.60	44.74 ± 13.05	40.87 ± 9.87	0.001 ^#^
Triglycerides. mg/dL	135.45 ±71.79	152.57 ± 78.23	132.61 ± 70.29	0.019 ^#^
Serum creatinine. mg/dL	0.96 ± 0.36	1.09 ± 0.44	1.05 ± 0.35	0.369 ^#^
Serum urea. mg/dL	44.71 ± 18.92	46.53 ± 21.77	44.43 ± 18.88	0.320 ^#^
Creatinine clearance. mL/min/1.73 m^2^	62.49 ± 22.01	61.71 ± 24.25	62.56 ± 21.51	0.721 ^#^
Fasting glucose. mg/dL	118.49 ± 48.90	117.90 ± 49.66	119.36 ± 49.22	0.787 ^#^
Serum fibrinogen. mg/dL	395.59 ± 132.22	393.21 ± 131.01	436.89 ± 133.11	0.049 ^#^
hs-CRP. mg/dL	6.34 ± 2.78	7.99 ± 2.91	5.03 ± 1.86	0.041 ^#^
Hematocrit. %	41.74 ± 5.16	40.95 ± 6.65	41.82 ± 5	0.132 ^#^
Platelets (×10^3^/mL)	297.44 ± 11.17	309.09 ± 99.95	296 ± 112.49	0.240 ^#^
**Clinical parameters**				
Pain at rest	541 (81.44%)	77 (77.0%)	464 (84.06%)	0.190 ^†^
Erythema	77 (11.81%)	12 (12.0%)	487 (88.22%)	0.949 ^†^
Ulcerations	93 (14.26%)	16 (16.0%)	77 (13.95%)	0.589 ^†^
Necrosis	27 (4.14%)	5 (5.0%)	22 (3.99%)	0.845 ^†^
Gangrene	121 (18.51%)	17 (17.0%)	103 (18.66%)	0.780 ^†^
Bilateral clinical involvement	231 (35.43%)	33 (33.0%)	198 (35.87%)	0.552 ^†^
Cardiac murmurs	119 (18.25%)	27 (27.0%)	92 (16.67%)	0.042 ^†^
Femoral artery murmur	149 (22.85%)	25 (25.0%)	124 (22.46%)	0.786 ^†^
Carotid artery murmur	77 (11.81%)	20 (20.0%)	57 (10.33%)	0.021 ^†^
Renal artery murmur	24 (3.68%)	8 (8.0%)	16 (2.90%)	0.041 ^†^
Ankle brachial index	0.81 ± 0.08	0.73 ± 0.11	0.82 ± 0.15	0.053 ^†^
**Paraclinical data**				
Arterial Doppler US	110 (16.95%)	21 (21.0%)	89 (16.12%)	
Angio MRI	32 (4.9%)	4 (4.0%)	28 (5.07%)	
Arteriography	635 (97.4%)	98 (98.0%)	536 (97.10%)	
Number of lesions (stenosis and thrombosis)				0.037 ^†^
0	5 (0.8%)	1 (1.0%)	4 (0.72%)	
1	226 (34.7%)	35 (35.0%)	191 (34.60%)	
2	183 (28.1%)	27 (27.0%)	156 (28.26%)	
3	98 (15.0%)	13 (13.0%)	85 (15.40%)	
4	65 (10.0%)	8 (8.0%)	57 (10.33%)	
5	40 (6.1%)	9 (9.0%)	31 (5.62%)	
≥6	35 (4.6%)	7 (7.0%)	28 (5.07%)	
LVEF, %	57.36 ± 10.08	57.30 ± 10.48	57.26 ± 10.05	0.973 ^#^
**Therapeutic management**				
Medical	650 (99.8%)	99 (99.0%)	551 (99.82%)	0.129 ^†^
Interventional revascularization	48 (7.36%)	5 (5.0%)	43 (7.79%)	0.326 ^†^
Surgical revascularization	369 (56.6%)	48 (48.0%)	320 (57.97%)	0.149 ^†^
Risk of amputation	210 (32.1%)	33 (33.0%)	177 (32.07%)	0.854 ^†^

All numerical values are expressed as mean ± standard deviation (SD). LDL-cholesterol: low-density lipoprotein cholesterol; HDL-cholesterol: high-density lipoprotein cholesterol; hs-CRP: high sensitivity C reactive protein (normal range 0–1 mg/dL); HbA1C: glycated hemoglobin; HR: heart rate; BP: blood pressure; ABI: ankle–brachial index; US: ultrasonography; MRI: magnetic resonance imaging; LVEF: left ventricle ejection fraction. * *p* value calculated by reference to BMI values of patients with varying degrees of obesity or overweight. ^#^ *p* value calculated using independent *t* test; ^†^ *p* value calculated using Mann–Whitney *U* test.

**Table 2:** Correlations between inflammatory markers and demographic, anthropometric or clinical–paraclinical parameters.

	Women				Men			
	Number of Stenotic Lesions and Thromboses		ABI		Number of Stenotic Lesions and Thromboses		ABI	
	r	*p* *	r	*p* *	r	*p*	r	*p* *
Age	−0.061	0.546	−0.059	0.566	−0.058	0.117	0.049	0.258
**Smoking (packs/year)**	**0.651**	**0.005**	**−0.400**	**<0.001**	**0.599**	**0.008**	**0.418**	**0.042**
Total cholesterol	0.048	0.638	0.046	0.656	0.072	0.092	0.009	0.828
HDL-cholesterol	0.136	0.176	−0.058	0.570	−0.11	0.791	0.060	0.167
LDL-cholesterol	0.042	0.677	0.039	0.705	0.050	0.244	−0.007	0.880
Triglycerides	−0.082	0.420	0.083	0.417	**0.100**	**0.019**	0.007	0.870
Systolic BP	−0.004	0.967	0.002	0.981	0.031	0.461	0.018	0.686
**Uric acid**	**0.351**	**<0.001**	0.097	0.345	0.019	0.650	−0.066	0.130
**hs-CRP**	**0.408**	**0.018**	**0.511**	**0.009**	**0.703**	**0.025**	**0.560**	**0.019**
**Serum fibrinogen**	**0.478**	**0.029**	**0.478**	**0.017**	**0.551**	**0.037**	**0.603**	**0.039**
BMI	0.010	0.923	0.060	0.562	−0.065	0.129	**−0.098**	**0.025**
More than 3 CVD risk factors	0.047	0.644	−0.087	0.398	**−0.146**	**0.001**	0.056	0.200

r: Pearson correlation; LDL: low-density lipoproteins; HDL: high-density lipoprotein; BP: blood pressure; BMI: body mass index; hs-CRP: high-sensitivity C reactive protein; CVD: cardiovascular disease; * *p* value calculated using Pearson correlation for all parameters with exception of the last one (more than 3 CVD risk factors).

**Table 3:** Predictors of amputation in patients with PAD–ROC analysis results.

Area Under the Curve					
Test Result Variable(s)	Area	Std. Error	Asymptotic Sig.	Asymptotic 95% Confidence Interval	
				Lower Bound	Upper Bound
Pulse pressure	0.545	0.025	0.062	0.497	0.594
Serum fibrinogen	0.639	0.024	<0.001	0.592	0.685
Gangrene	0.754	0.023	<0.001	0.709	0.799
Men	0.636	0.021	<0.001	0.594	0.678
HDL-cholesterol < 40 mg/dL	0.640	0.023	<0.001	0.593	0.686
More than 3 CVD risk factors	0.679	0.022	<0.001	0.635	0.723
Smoking	0.838	0.016	<0.001	0.808	0.869
ABI ≤ 0.5	0.787	0.019	<0.001	0.750	0.823
Uncontrolled diabetes mellitus	0.679	0.024	<0.001	0.631	0.726
Pain at rest	0.614	0.022	<0.001	0.571	0.657

HDL: high density lipoprotein; CVD: cardiovascular disease; ABI: ankle–brachial index.

**Table 4:** Predictors of amputation in patients with PAD-univariate and multivariate statistical analysis.

Parameter	Univariate Regression			Multivariate Regression		
	*β*	*p*	Odds Ratio (95% CI)	*β*	*p*	Odds Ratio (95% CI)
Serum fibrinogen	0.004	<0.001	1.004 (1.003–1.005)	−0.003	0.045	0.997 (0.969–1.024)
Gangrene	3.880	<0.001	8.436 (1.466–25.892)	3.752	<0.001	8.206 (2.687–17.105)
Men	3.009	<0.001	6.559 (3.761–9.367)	2.789	<0.001	6.313 (3.115–8.006)
HDL-cholesterol less than 40 mg/dL	1.164	<0.001	3.202 (2.277–4.503)	1.393	<0.001	2.948 (1.194–4.882)
More than 3 CVD risk factors	1.489	<0.001	4.434 (3.106–6.330)	2.364	<0.001	4.138 (3.040–7.225)
Smoking	4.265	<0.001	11.141 (5.579–15.345)	4.679	<0.001	10.660 (5.874–27.956)
ABI ≤ 0.5	2.893	<0.001	18.054 (11.119–29.313)	3.666	<0.001	16.282 (9.182–30.821)
Uncontrolled diabetes mellitus	2.089	<0.001	4.080 (5.304–12.307)	1.342	0.161	3.826 (0.586–25.004)
Pain at rest	3.093	<0.001	12.039 (2.906–17.327)	3.489	0.006	12.168 (2.711–16.085)

HDL: high density lipoprotein; CVD: cardiovascular disease; ABI: ankle–brachial index.

## Data Availability

All the data are available from the corresponding author upon reasonable request.
